# Family history and gastric cancer incidence and mortality in Asia: a pooled analysis of more than half a million participants

**DOI:** 10.1007/s10120-024-01499-1

**Published:** 2024-04-22

**Authors:** Dan Huang, Minkyo Song, Sarah Krull Abe, Md. Shafiur Rahman, Md. Rashedul Islam, Eiko Saito, Katherine De la Torre, Norie Sawada, Akiko Tamakoshi, Xiao-Ou Shu, Hui Cai, Atsushi Hozawa, Seiki Kanemura, Jeongseon Kim, Yu Chen, Hidemi Ito, Yumi Sugawara, Sue K. Park, Myung-Hee Shin, Mayo Hirabayashi, Takashi Kimura, Yu-Tang Gao, Wanqing Wen, Isao Oze, Aesun Shin, Yoon-Ok Ahn, Habibul Ahsan, Paolo Boffetta, Kee Seng Chia, Keitaro Matsuo, You-Lin Qiao, Nathaniel Rothman, Wei Zheng, Manami Inoue, Daehee Kang

**Affiliations:** 1 Department of Preventive Medicine, Seoul National University College of Medicine, 103 Daehak-Ro, Jongno-Gu, Seoul, 03080 Korea; 2 Integrated Major in Innovative Medical Science, Seoul National University Graduate School, Seoul, Korea; 3 Infections and Immunoepidemiology Branch, Division of Cancer Epidemiology and Genetics, National Cancer Institute, Baltimore, MD USA; 4 Laboratory of Epidemiology and Population Sciences, National Institute on Aging, National Institute of Health, Bethesda, MD USA; 5 Division of Prevention, National Cancer Center Institute for Cancer Control, Tokyo, Japan; 6 Research Center for Child Mental Development, Hamamatsu University School of Medicine, Tokyo, Japan; 7 Hitotsubashi Institute for Advanced Study, Hitotsubashi University, Tokyo, Japan; 8 Institute for Global Health Policy Research, National Center for Global Health and Medicine, Tokyo, Japan; 9 Department of Biomedical Sciences, Seoul National University Graduate School, Seoul, Korea; 10 Division of Cohort Research, National Cancer Center Institute for Cancer Control, Tokyo, Japan; 11 Department of Public Health, Hokkaido University Faculty of Medicine, Sapporo, Japan; 12 Division of Epidemiology, Department of Medicine, Vanderbilt Epidemiology Center, Vanderbilt-Ingram Cancer Center, Vanderbilt University Medical Center, Nashville, TN USA; 13 Tohoku University Graduate School of Medicine, Sendai, Miyagi Prefecture Japan; 14 Graduate School of Cancer Science and Policy, National Cancer Center, Goyang, Korea; 15 Departments of Population Health and Environmental Medicine, NYU Grossman School of Medicine, New York, NY USA; 16 Division of Cancer Information and Control, Department of Preventive Medicine, Aichi Cancer Center Research Institute, Nagoya, Japan; 17 Division of Descriptive Cancer Epidemiology, Nagoya University Graduate School of Medicine, Nagoya, Japan; 18 Department of Social and Preventive Medicine, Sungkyunkwan University School of Medicine, Seoul, Korea; 19 Department of Epidemiology, Shanghai Cancer Institute, Shanghai, China; 20 Renji Hospital, Shanghai Jiaotong University School of Medicine, Shanghai, China; 21 Division of Cancer Epidemiology and Prevention, Aichi Cancer Center Research Institute, Nagoya, Japan; 22 Cancer Research Institute, Seoul National University, Seoul, Korea; 23 Department of Preventive Medicine, Seoul National University College of Medicine, Seoul, Korea; 24 Department of Public Health Sciences, University of Chicago, Chicago, IL USA; 25 Stony Brook Cancer Center, Stony Brook University, Stony Brook, NY USA; 26 Department of Medical and Surgical Sciences, University of Bologna, Bologna, Italy; 27 Saw Swee Hock School of Public Health, National University of Singapore, Singapore, Singapore; 28 Department of Cancer Epidemiology, Nagoya University Graduate School of Medicine, Nagoya, Japan; 29 School of Population Medicine and Public Health, Chinese Academy of Medical Sciences and Peking Union Medical College, Beijing, China; 30 Division of Cancer Epidemiology and Genetics, Occupational and Environmental Epidemiology Branch, National Cancer Institute, Bethesda, MD USA; 31 Division of Epidemiology, Department of Medicine, Vanderbilt Epidemiology Center, Vanderbilt University Medical Center, Nashville, TN USA

**Keywords:** Gastric cancer, Family history, Incidence, Mortality, Asian

## Abstract

**Background:**

The family history of gastric cancer holds important implications for cancer surveillance and prevention, yet existing evidence predominantly comes from case–control studies. We aimed to investigate the association between family history of gastric cancer and gastric cancer risk overall and by various subtypes in Asians in a prospective study.

**Methods:**

We included 12 prospective cohorts with 550,508 participants in the Asia Cohort Consortium. Cox proportional hazard regression was used to estimate study-specific adjusted hazard ratios (HRs) and 95% confidence intervals (CIs) for the association between family history of gastric cancer and gastric cancer incidence and mortality, then pooled using random-effects meta-analyses. Stratified analyses were performed for the anatomical subsites and histological subtypes.

**Results:**

During the mean follow-up of 15.6 years, 2258 incident gastric cancers and 5194 gastric cancer deaths occurred. The risk of incident gastric cancer was higher in individuals with a family history of gastric cancer (HR 1.44, 95% CI 1.32–1.58), similarly in males (1.44, 1.31–1.59) and females (1.45, 1.23–1.70). Family history of gastric cancer was associated with both cardia (HR 1.26, 95% CI 1.00–1.60) and non-cardia subsites (1.49, 1.35–1.65), and with intestinal- (1.48, 1.30–1.70) and diffuse-type (1.59, 1.35–1.87) gastric cancer incidence. Positive associations were also found for gastric cancer mortality (HR 1.30, 95% CI 1.19–1.41).

**Conclusions:**

In this largest prospective study to date on family history and gastric cancer, a familial background of gastric cancer increased the risk of gastric cancer in the Asian population. Targeted education, screening, and intervention in these high-risk groups may reduce the burden of gastric cancer.

**Supplementary Information:**

The online version contains supplementary material available at 10.1007/s10120-024-01499-1.

## Introduction

Despite the incidence and mortality of gastric cancer having declined globally in the past decades, gastric cancer remains one of the most common cancers worldwide with more than one million new cases and nearly 800,000 deaths every year [1]. The majority of gastric cancers occur in Asia, with Eastern Asia accounting for more than 60% of new cases with the highest incidence and mortality rates in the world [1, 2]. Family history of gastric cancer has been shown as an important risk for gastric cancer and can be used as a simple tool to risk-stratify individuals for further evaluation in countries where there are limited resources for population screening [3–6].

The familial clustering of gastric cancer might be interpreted through several mechanisms, including hereditary and nonhereditary factors [7]. Shared environmental factors, such as *Helicobacter pylori* infection, smoking, dietary intake, and socioeconomic status, may by themselves or have synergistic effects in individuals with genetic predisposition [8–10]. Identifying individuals at high risk of the disease is important for gastric cancer surveillance and prevention.

The findings on first-degree family history of gastric cancer and the risk of gastric cancer mostly come from case–control studies [11–13], which are inherently prone to recall bias. Furthermore, it remains unclear whether different types of family history of gastric cancer (a parent or a sibling) is associated with gastric cancer risk differently. It is also uncertain whether a family history of gastric cancer is related to the risk of specific subtypes of gastric cancer defined by anatomical subsites or histology. In the Asian Cohort Consortium, we aimed to investigate the association between a family history of gastric cancer and risk of developing gastric cancer, both in terms of overall risk and specific subtypes, within the Asian populations.

## Methods

### Study population

We conducted pooled analyses using data from population-based cohort studies participating in the Asia Cohort Consortium (ACC) [14, 15]. The ACC is a collaborative effort that includes 44 cohorts from 10 Asian countries, seeking to understand the relationship between genetics, environmental exposures, and the etiology of diseases including cancer, through the establishment of a cohort of at least one million healthy people [16]. The current pooled analysis includes 12 cohort studies with information on family history, gastric cancer incidence and/or mortality data, conducted in Japan (*N* = 330,303), China (*N* = 164,277), and Korea (*N* = 55,928). The participating cohorts include Three Prefecture Cohort Study Miyagi (Miyagi3p), Three Prefecture Cohort Study Aichi (Aichi3p), Japan Collaborative Cohort Study (JACC), Miyagi Cohort Study (Miyagi), Japan Public Health Center-based prospective Study I and II (JPHC I/II), Ohsaki National Health Insurance Cohort Study (Ohsaki), Linxian General population Trial Cohort (Linxian), Shanghai Women’s Health Study (SWHS), Shanghai Men’s Health Study (SMHS), Korean Multi-center Cancer Cohort Study (KMCC), and Korean National Cancer Center Cohort (KNCC) (Supplementary Table 1). Among 628,879 participants, we excluded those who had no or inaccurate information on age and/or sex (*n* = 532), follow-up (*n* = 18,217), gastric cancer incidence (*n* = 51,234), or family history of gastric cancer (*n* = 65,124), and those who had a prior cancer diagnosis at baseline (*n* = 10,180). The final gastric cancer incidence analytic sample included 550,508 participants (Supplementary Table 2). For the mortality analyses, we excluded those who had no or inaccurate information on age and/or sex (*n* = 532), follow-up (*n* = 18,221), and family history of gastric cancer (*n* = 65,124), after which we had 531,137 participants for the final mortality analytic set (Supplementary Table 3).

### Assessment of family history of gastric cancer

Each study assessed the information on family history of gastric cancer by self-administered questionnaire at enrollment. Seven cohorts had detailed information on the relationship and sex of the affected family member. Family history of gastric cancer was defined as those with a first-degree relative (parent or sibling) being affected. We assessed family history of gastric cancer in parents (father or mother) and siblings (brother or sister) separately, and considered sex concordance between study subjects and their affected family members.

### Outcome assessment

Incident gastric cancers were identified by linkage to national cancer registries or self-reports confirmed by medical record review in each cohort. The International Classification of Diseases, 9th and 10th revision (ICD-9 151.1–151.9; ICD-10 C16.0–16.9) codes were used for the identification of gastric cancer. The primary outcome was defined as the first occurrence of gastric cancer. The anatomic subsites of gastric cancer were classified as cardia (ICD-9 151.0; ICD-10 C16.0) and non-cardia (ICD-9 151.1–151.6; ICD-10 C16.1–16.6). Using ICD for Oncology 3, gastric cancer histological subtypes were assessed as intestinal- (8012, 8021, 8022, 8031, 8032, 8046,8050, 8082, 8143, 8144, 8201, 8210, 8211, 8220, 8221, 8255, 8260, 8261, 8262, 8263, 8310, 8323, 8480, 8481, 8510, 8512, 8570, 8576), or diffuse- (8020, 8041, 8044, 8141, 8142, 8145, 8490, 8806) types [17]. Gastric cancer mortality was defined as death due to gastric cancer (ICD-9 151 and ICD-10 C16) during follow-up.

### Statistical analyses

The follow-up was until the date of gastric cancer incidence (or death), loss to follow-up, or the last date of follow-up of each cohort, whichever occurred first. Cox proportional hazard regression models were used to calculate the hazard ratios (HRs) and their corresponding 95% confidence intervals (CIs) of the incidence or mortality of gastric cancer associated with family history of gastric cancer, with age as the time metric. A non-proportional test using Schoenfeld partial residuals to evaluate whether the Cox proportional hazards assumption holds for predictors across different time periods with each cohort. We included sex, cigarette smoking status (never/ever), alcohol consumption status (never/ever), education level (low/high: no formal education or primary education classified as low education level, secondary education or above classified as high education level), and body mass index (BMI, continuous kg/m^2^) as covariates in the models, depending on the availability in each cohort (Supplementary Table 1). For the missing values, we assigned the cohort-specific median (for continuous) or percentage of high-dimensional values (for categorical) of the non-missing covariates. The summary effect estimates were computed by pooling study-specific HRs using random-effects models. In addition, we performed stratified analyses by country (Japan, South Korea, China), sex (male and female), cohort enrollment period (the 80s, 90s, 90–00s, and 00s), cohort enrollment age (< 55 and ≥ 55), birth year (< 1925, 1925–1940, and ≥ 1940), education level (low and high), smoking status (never and ever), and alcohol consumption status (never and ever) to evaluate effect modifications. Heterogeneity between studies or stratified subgroups was evaluated using the *Q* statistics and quantified using *I*^2^. All statistical analyses were performed using SAS (version 9.4) and STATA software (version 16.0). The two-tailed *p* < 0.05 was considered to be statistically significant.

## Results

Table 1 shows the characteristics of the 12 cohorts for the analysis of family history of gastric cancer and gastric cancer incidence. During the median follow-up of 15.6 years, 12,258 gastric cancer cases occurred. Among the study participants, 46.1% of participants were male, 37.5% were ever smokers, 41.3% were ever drinkers, 27.0% with low education levels, and 8.9% had a family history of gastric cancer at baseline enrollment.

**Table 1:** Characteristics of 12 cohort studies in the Asia Cohort Consortium included in the pooled analysis of family history of gastric cancer and gastric cancer incidence (*N* = 550,508)

Cohort name	Participants, *N*	Period of enrollment, years	Years of follow-up, mean (SD)	Age at enrollment, mean (SD)	Body mass index, kg/m^2^, mean (SD)	Male, %	Ever smoking, %	Ever drinking, %	Low education^a^, %	Family history of gastric cancer, %	Incident gastric cancer^b^, *N*
Japan											
Miyagi3P	30,809	1984	7.6 (2.6)	57.2 (11.3)	23.3 (3.4)	44.6	30.6	47.3	NA	11.7	491
Aichi3P	24,917	1985	11.5 (5.1)	55.4 (10.9)	22.1 (3.0)	47.6	47.4	61.4	NA	10.0	442
JACC	81,494	1988–1990	16.1 (5.7)	57.5 (10.0)	22.8 (3.6)	42.1	35.5	46.7	17.9	11.2	1429
Miyagi	46,132	1990	21.5 (6.0)	52.0 (7.5)	23.6 (3.0)	48.2	42.4	52.2	54.8	10.6	1940
JPHC	42,136	1990–1992	21.0 (4.3)	49.5 (5.9)	23.6 (3.0)	48.2	40.3	50.1	49.6	6.8	1007
JPHC2	55,317	1992–1995	17.7 (4.2)	54.2 (8.8)	23.5 (3.1)	47.7	40.0	49.1	NA	5.9	1351
Ohsaki	49,498	1995	10.8 (4.3)	60.2 (10.3)	23.6 (3.3)	48.1	41.3	49.8	56.0	13.3	1350
China											
Linxian	29,458	1984–1987	21.3 (10.9)	52.8 (8.9)	22.0 (2.5)	44.6	30	23.9	96.2	3.5	2508
SWHS	73,355	1996–2000	17. 4 (2.9)	52.5 (9.1)	24.0 (3.4)	0	2.8	2.3	21.4	5.8	452
SMHS	61,464	2001–2006	11.9 (2.4)	55.4 (9.7)	23.7 (3.1)	100	69.6	33.7	6.6	6.4	562
South Korea											
KMCC	20,323	1993–2005	13.9 (4.6)	54.0 (14.3)	23.6 (3.3)	40.0	36.2	41.1	72.4	4.8	430
KNCC	35,605	2007–2015	9.3 (3.3)	49.7 (9.2)	23.9 (3.0)	51.4	33.5	67.7	6.8	13.6	296
Total (12)	550,508		15.6 (6.4)	54.5 (10.0)	23.4 (3.2)	46.1	37.5	41.3	27.0	8.9	12,258

*N* number, *SD* standard deviation, *NA* not available, *Miyagi3p* Three Prefecture Cohort Study Miyagi, *Aichi3p* Three Prefecture Cohort Study Aichi, *JACC* Japan Collaborative Cohort Study, *Miyagi* Miyagi Cohort Study, *JPHC* Japan Public Health Center-based prospective Study, *Ohsaki* Ohsaki National Health Insurance Cohort Study, *Linxian* Linxian General population Trial Cohort, *SWHS* Shanghai Women’s Health Study, *SMHS* Shanghai Men’s Health Study, *KMCC* Korean Multi-center Cancer Cohort Study, *KNCC* Korean National Cancer Center Cohort

^a^Primary school education or below

^b^First primary gastric cancer cases

In most cohorts, individuals with family history of gastric cancer were at increased risk compared to those without family history of gastric cancer, with the risk estimate ranging from HR 1.03 (Linxian) to 1.99 (KMCC; Table 2). The pooled HR was 1.44 (95% CI 1.32–1.58) with a significant heterogeneity of the association across the cohorts (*p*_heterogeneity_ = 0.006).

**Table 2:**
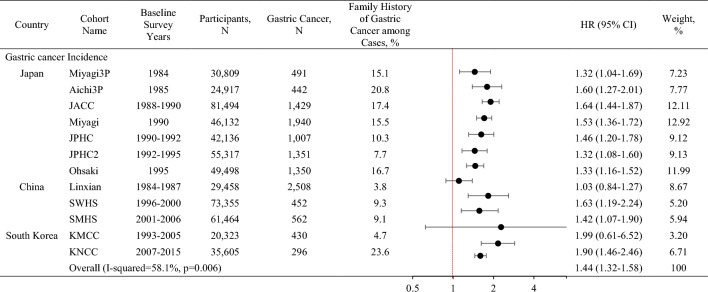
Association between family history of gastric cancer and gastric cancer risk in the Asia Cohort Consortium

*Miyagi3p* Three Prefecture Cohort Study Miyagi, *Aichi3p* Three Prefecture Cohort Study Aichi, *JACC* Japan Collaborative Cohort Study, *Miyagi* Miyagi Cohort Study, *JPHC* Japan Public Health Center-based prospective Study, *Ohsaki* Ohsaki National Health Insurance Cohort Study, *Linxian* Linxian General population Trial Cohort, *SWHS* Shanghai Women’s Health Study, *SMHS* Shanghai Men’s Health Study, *KMCC* Korean Multi-center Cancer Cohort Study, *KNCC* Korean National Cancer Center Cohort, *N* number, *HR* Hazard ratio

Hazard ratios (HR) and 95% confidence intervals (CI) calculated adjusted for sex, smoking (ever/never), alcohol consumption (ever/never), education (low/high), and body mass index (kg/m^2^, continuous) depending on availability in each cohort

Table 3 shows the association between family history of gastric cancer and gastric cancer incidence by various groups. By country, a positive association was shown in Japan (HR 1.47, 95% CI 1.37–1.57) but not in China (1.31, 0.99–1.75) or South Korea (1.50, 0.90–2.51) where there was substantial heterogeneity among studies within each country (*I*^2^ = 70.6% vs. 75.7%, respectively). The associations were similar in males (HR 1.44, 95% CI 30–1.58) and females (1.45, 1.23–1.70; *p*_heterogeneity_ = 0.94). The association between family history of gastric cancer and gastric cancer incidence was stronger in individuals with younger age at enrollment (< 55 years old: HR 1.64, 95% CI 1.44–1.86), than that in those enrolled at a later age (≥ 55 years old: 1.35, 1.21–1.51; *p*_heterogeneity_ = 0.01). Similar associations were observed by enrollment year (1980–1989: HR 1.38, 95% CI 1.11–1.72, 1990–1999: 1.43, 1.33–1.54, after 2000: 1.50, 1.13–2.00), birth year (< 1925: 1.26, 1.01–1.57, 1925–1940: 1.43, 1.25–1.64, ≥ 1940: 1.63, 1.43–1.85), age at diagnosis (< 60 years old: 1.65, 1.42–1.92, 60–70: 1.36, 1.17–1.58, ≥ 70: 1.35, 1.14–1.61), and education level (low: 1.36, 1.23–1.52, high: 1.51, 1.35–1.68).

**Table 3:** Association between family history of gastric cancer and gastric cancer incidence by selected subgroups

Subgroup	Studies, *N*	Participants, *N*	Gastric cancer, *N*	Family history of gastric cancer among cases, %	HR (95% CI)^a^	Heterogeneity within subgroups^b^		Heterogeneity between subgroups^c^
						*I*^2^ value (%)	*p*-value	
Country								0.74
Japan	7	330,303	8010	14.3	1.47 (1.37–1.57)	35.9	0.15	
China	3	164,277	3522	5.3	1.31 (0.99–1.75)	70.6	0.03	
South Korea	2	55,928	729	12.3	1.50 (0.90–2.51)	75.7	0.04	
Sex								0.94
Male	11	253,668	8156	11.5	1.44 (1.30–1.58)	48.7	0.03	
Female	11	296,840	4102	11.9	1.45 (1.23–1.70)	60.9	< 0.01	
Enrollment years								0.52
1980–1989	4	166,678	4870	10.5	1.38 (1.11–1.72)	82.2	< 0.01	
1990–1999	5	266,438	6100	12.7	1.43 (1.33–1.54)	0	0.42	
After 2000	3	117,392	1288	20.3	1.50 (1.13–2.00)	57.9	0.09	
Age at enrollment, years								0.01
< 55	12	290,294	4444	6.6	1.64 (1.44–1.86)	35.0	0.11	
≥ 55	12	260,214	7814	11.9	1.35 (1.21–1.51)	39.8	0.08	
Birth year								0.14
< 1925	6	139,642	4820	9.5	1.26 (1.01–1.57)	79.5	< 0.01	
1925–1940	10	171,920	5025	13.0	1.43 (1.25–1.64)	43.9	0.07	
≥ 1940	10	238,946	2413	13.0	1.63 (1.43–1.85)	17.3	0.28	
Age at diagnosis, years								0.12
< 60	12	91,335	2498	12.5	1.65 (1.42–1.92)	39.7	0.08	
60–70	12	272,597	6976	11.2	1.36 (1.17–1.58)	58.9	0.01	
≥ 70	12	186,576	2784	11.2	1.35 (1.14–1.61)	42.3	0.06	
Education level^d^								0.21
Low	8	139,052	5546	9.1	1.36 (1.23–1.52)	37.0	0.13	
High	7	206,661	2636	13.7	1.51 (1.35–1.68)	0	0.61	

*N* number, *HR* hazard ratio, *CI* confidence interval

^a^HR refers to a summary estimate of effects based on a random-effects model, adjusted for by sex, smoking (ever/never), alcohol consumption (ever/never), education (low/high) and body mass index (kg/m^2^, continuous) depending on availability in each cohort

^b^*p* value for heterogeneity within each group

^c^*p* value for heterogeneity between subgroups

^d^Primary school education or below

In the stratified analyses by different gastric cancer subtypes, the positive association between family history of gastric cancer and gastric cancer risk was similar by different anatomical subsites (cardia: HR 1.26, 95% CI 1.00–1.60, non-cardia: 1.49, 1.35–1.65; *p*_heterogeneity_ = 0.35) or histological subtypes (intestinal: 1.48, 1.30–1.70, diffuse: 1.59, 1.35–1.87; *p*_heterogeneity_ = 0.50; Table 4). When analyzed separately by sex, there was no difference in the family history of gastric cancer association based on anatomical subsite or histological subtype, both among males and females.

**Table 4:** Association between family history of gastric cancer and gastric cancer incidence by gastric cancer subtypes

Characteristics	Studies, *N*	Participants, *N*	Gastric cancer, *N*	Family history of gastric cancer among cases, %	HR (95% CI)^a^	Heterogeneity within subgroups^b^		Heterogeneity between subgroups^c^
						*I*^2^ value (%)	*p*-value	
Total								
Anatomical subsite^d^								0.35
Cardia	8	538,220	634	12.5	1.26 (1.00–1.60)	0	0.92	
Non-cardia	9	545,086	4980	14.3	1.49 (1.35–1.65)	56.5	0.02	
Histological subtype^e^								0.50
Intestinal	4	538,971	1385	12.3	1.48 (1.30–1.70)	20.4	0.29	
Diffuse	4	537,870	284	14.4	1.59 (1.35–1.87)	0	0.55	
Male								
Anatomical subsite^d^								0.85
Cardia	8	245,494	511	13.5	1.42 (1.10–1.83)	0	0.91	
Non-cardia	9	248,401	3418	13.2	1.46 (1.29–1.66)	28.2	0.20	
Histological subtype^e^								0.93
Intestinal	4	246,018	1035	11.2	1.50 (1.16–1.94)	37.6	0.19	
Diffuse	4	245,125	142	12.0	1.54 (1.02–2.58)	0	0.86	
Female								
Anatomical subsite^d^								0.07
Cardia	8	292,726	123	8.1	0.87 (0.45–1.67)	0	0.96	
Non-cardia	9	294,165	1562	15.5	1.63 (1.37–1.93)	26.3	0.21	
Histological subtype^e^								0.82
Intestinal	4	292,953	350	15.7	1.92 (1.44–2.57)	0	0.78	
Diffuse	4	292,745	142	16.9	1.72 (1.09–4.33)	62.2	0.05	

*N* number, *HR* hazard ratio, *CI* confidence interval

^a^HR refers to a summary estimate of effects based on a random-effects model, adjusted for by sex, smoking (ever/never), alcohol consumption (ever/never), education (low/high) and body mass index (kg/m^2^, continuous) depending on availability in each cohort

^b^*p* value for heterogeneity within each group

^c^*p* value for heterogeneity between subgroups

^d^Anatomical subsites by International Classification of Diseases (ICD); cardia (ICD-9: 151.0, ICD-10: C16.0), non-cardia (ICD-9: 151.1–151.6, 151.8–151.9, ICD-10: C16.1–16.6, 16.8–16.9)

^e^Lauren classification by ICD for Oncology 3: intestinal type (8012, 8021, 8022, 8031, 8032, 8046,8050, 8082, 8143, 8144, 8201, 8210, 8211, 8220, 8221, 8255, 8260, 8261, 8262, 8263, 8310, 8323, 8480, 8481, 8510, 8512, 8570, and 8576), diffuse type (8020, 8041, 8044, 8141, 8142, 8145, 8490, and 8806)

The risk of gastric cancer was similarly high in individuals with a family history of gastric cancer in parents (HR 1.45, 95% CI 1.23–1.71) and siblings (1.47, 1.28–1.70; Table 5) (*p*_heterogeneity_ = 0.90). Specifically, the magnitude of associations was not statistically different across affected family members, showing a range between HR of 1.23 (sister) to 1.80 (brother). When stratified by sex of the study participant, in males, having a family history of gastric cancer in a brother was most strongly associated with gastric cancer risk (HR 1.94, 95% CI 1.23–3.07), followed by affected fathers (1.57, 1.17–2.10) and mothers (1.49, 1.12–1.98). There was no statistical association with affected sisters (1.36, 0.73–2.53) compare to those without affected family members. There was no statistical heterogeneity between these associations. In females, the positive significant association was only shown in those with affected mothers (HR 1.82, 95% CI 1.02–3.24), but not in those with affected fathers (1.75, 0.99–3.10) (*p*_heterogeneity_ = 0.93).

**Table 5:** Association between family history of gastric cancer and gastric cancer incidence by relationships to the affected family member

Subgroup	Studies, *N*	Participants, *N*	Gastric cancer, *N*	HR (95%CI)^a^	Heterogeneity within subgroups^b^		Heterogeneity between subgroups^c^
					*I*^2^ value (%)	*p*-value	
Family relations							0.90
Parents	3	135,067	172	1.45 (1.23–1.71)	0	0.65	0.87
Father	3	131,957	114	1.59 (1.32–1.92)	0	0.51	
Mother	3	129,065	58	1.55 (1.21–1.99)	23.1	0.27	
Sibling	3	128,121	48	1.47 (1.28–1.70)	0	0.46	0.29
Brother	3	127,298	35	1.80 (1.23–2.62)	0	0.27	
Sister	2	126,582	11	1.23 (0.68–2.23)	0	0.91	
Family relations by participant’s sex							
Male participant							0.76
Father	3	95,488	84	1.57 (1.17–2.10)	43.5	0.17	
Mother	3	93,610	44	1.49 (1.12–1.98)	0	0.81	
Brother	3	92,259	28	1.94 (1.23–3.07)	33.1	0.22	
Sister	2	91,785	10	1.36 (0.73–2.53)	0	0.75	
Female participant							0.93
Father	2	36,469	30	1.75 (0.99–3.10)	48.8	0.16	
Mother	2	35,455	14	1.82 (1.02–3.24)	18.8	0.27	

*N* number, *HR* hazard ratio, *CI* confidence interval

Results of gastric cancer with less than 10 cases are not shown in the table

^a^HR refers to a summary estimate of effects based on a random-effects model, adjusted for by sex, smoking (ever/never), alcohol consumption (ever/never), education (low/high) and body mass index (kg/m^2^, continuous) depending on availability in each cohort

^b^*p* value for heterogeneity within each group

^c^*p* value for heterogeneity between subgroups

We further examined the association between family history of gastric cancer (9.2% at baseline) and gastric cancer mortality (*n* = 5194) in a total of 531,137 subjects from 11 cohorts (Supplementary Table 4). Similar to incidence, family history of gastric cancer was positively associated with gastric cancer mortality (HR 1.31, 95% CI 1.20–1.42; Supplementary Tables 5). The association did not differ appreciably by various subgroups (Supplementary Table 6). Similar associations were observed across the country (Japan: HR 1.31, 95% CI 1.20–1.43, China: 1.37, 1.07–1.76, South Korea: 0.77, 0.37–1.58), sex (males: 1.32, 1.19–1.47; females: 1.45, 1.27–1.67), enrollment year (1980–1989: 1.33, 1.17–1.52, 1990–1999: 1.28, 1.14–1.44, after 2000: 1.35, 0.97–1.88), age at enrollment (< 55 years old: 1.39, 1.21–1.59, ≥ 55 years old: 1.27, 1.15–1.40), birth year (< 1925: 1.27, 1.13–1.43, 1925–1940: 1.41, 1.20–1.65, ≥ 1940: 1.38, 1.16–1.64), and education level (low: 1.30, 1.11–1.53, high: 1.27, 1.05–1.52).

In the analysis stratified by gastric cancer subtypes (Supplementary Table 7), the positive association between family history of gastric cancer and gastric cancer mortality persisted regardless of anatomical subsites (cardia: HR 1.31, 95% CI 1.19–1.44; non-cardia: 1.39, 1.19–1.62), or histological subtypes (intestinal type: 1.55, 1.02–2.36, diffuse type: 1.17, 0.56–2.42).

The risk of gastric cancer mortality was similarly high in individuals with a family history of gastric cancer in parents (HR 1.30, 95% CI 1.00–1.70) and siblings (1.63, 1.05–2.55). The magnitude of association for gastric cancer mortality was highest in those with a brother with gastric cancer (HR 1.93, 95% CI 1.16–3.22), although not statistically different from others (Supplementary Table 8).

## Discussion

In a large prospective pooled analysis in the ACC, we confirmed that family history of gastric cancer is associated with increased risk of gastric cancer in the Asian population. While we did not observe a statistically significant difference, the magnitude of association was more pronounced among individuals who were born later years, or diagnosed with gastric cancer at a younger age. We found no difference in risk among histological subtypes of gastric cancer. Although not statistically different from other associations, in males, having a family history of gastric cancer among brothers was most strongly associated with gastric cancer, followed by those affected fathers and mothers, compared to those without affected family members. In females, a significant positive association was only observed in those with affected mothers. Similar to incidence, we confirmed positive associations between family history of gastric cancer and gastric cancer mortality.

Previous studies have suggested that gastric cancer exhibits a greater tendency toward familial clustering and a higher predisposition to the disease than other cancers [18]. Approximately 10% of gastric cancers display a familial aggregation [19] but only less than 3% of gastric cancer arise from hereditary gastric cancers [20]. Familial diffuse gastric cancer and hereditary diffuse gastric cancer are the most recognizable, familial gastric cancer caused by APC, CDH1, and CTNNA1 gene mutations, respectively [21]. Still, the frequency of hereditary diffuse gastric cancer is rare (incidence 0.3%–3.1% in South Korea and Japan) and does not account for a large proportion of familial clustering [22, 23]. Interestingly, according to the results of a recent investigation into germline variants in nine cancer genes and their association with gastric cancer risk, it appears that the role of hereditary factors in contributing to the likelihood of developing gastric cancer may be greater than previously thought [24]. In addition to genetic factors, family-shared environmental factors may play an important role by interacting with low penetrance genes. Several studies reported that *H. pylori* infection and precancerous tissue changes (such as atrophy and intestinal metaplasia) are more common in children and siblings of patients with gastric cancer [9, 25, 26]. Notably, a randomized trial in South Korea reported that among persons with *H. pylori* infection who had a family history of gastric cancer in first-degree relatives, *H. pylori* eradication treatment reduced the risk of gastric cancer [26]. Moreover, an individual’s dietary sodium intake and preference for salty foods may be influenced by their family environment [27]. Consuming a diet high in salt may increase the likelihood of contracting an *H. pylori* infection, and even worse, it can enhance the ability of *H. pylori* to promote the development of gastric cancer by boosting cagA expression [28], or directly damage the mucosal barrier [29]. 

Although the difference was statistically not significant, we observed that the association between family history of gastric cancer was more prominent in individuals diagnosed with non-cardia gastric cancers than in cardia gastric cancers [30, 31]. The stronger association in non-cardia gastric cancers have been observed in several studies in Asia [31] and also in other parts of the world [11, 32]. The reason for this difference is not entirely clear, but several factors may be involved. One possible explanation is that non-cardia gastric cancer and cardia gastric cancer may have different risk factors. Non-cardia gastric cancer is more strongly associated with chronic gastritis and *H. pylori* infection, while cardia gastric cancer is more strongly linked to obesity and gastroesophageal reflux disease [33, 34]. Another possible explanation is that the genetic factors contributing to the development of non-cardia gastric cancer may differ from those contributing to cardia gastric cancer. Studies have identified several genetic variants differentially associated with an increased risk of gastric cancer by anatomical subsite [35]. A combination of various environmental risks with genetic predisposition may synergistically affect one subsite than the other [24].

Due to the sex-specific differences in the incidence of gastric cancer, with males having twice the incidence of females, and potential behavioral risk factors such as alcohol consumption and smoking varying accordingly [36], we stratified our analyses by sex. We observed no sex differences in the association between family history of gastric cancer and risk of gastric cancer in the current study. Several earlier case–control studies reported higher association in females compared to males, which may reflect sex differences in recall bias in reporting the family history [37–39]. Our findings are consistent with recent studies from a large consortium of gastric cancer studies from 17 studies from 11 countries (5946 cases) [11], and a meta-analysis [32]. However, further research is needed to fully understand the underlying mechanisms behind these non-differential associations by sex despite the differences in the exposure and the outcome.

Individuals enrolled at a younger age had a stronger association between family history of gastric cancer and the risk of developing the disease than those enrolled later. In addition, the association was more pronounced among those diagnosed with gastric cancer at a younger age and born later. There are several potential explanations for these results, one possibility is that these individuals may have a stronger genetic predisposition to gastric cancer, which is compounded by a common environment shared within the family [40, 41].

Early-onset cancer, familial aggregation of cancer, and diagnosis of multiple primary tumors are all indicative of an inherited cancer predisposition [42, 43]. Gastric cancer is typically diagnosed after age 60, but when it occurs before age 50, there is often a familial predisposition [44, 45]. This suggests that genetic factors may play a stronger role in the development of gastric cancer at a younger age. Another factor that could contribute to the more substantial genetic component in young individuals with gastric cancer is the interaction between genetic factors and additional risk factors accumulated over time. Unhealthy lifestyle habits or dietary patterns may interact with genetic predispositions to increase cancer risk [46]. Finally, younger individuals may be more likely to undergo regular screenings and have a higher awareness of the diseases when they have close family members who have been diagnosed with gastric cancer [47, 48].

In this study, our findings confirm that a family history of gastric cancer is associated with an elevated risk of gastric cancer within the Asian population. While the incidence rate of gastric cancer among individuals with a family history is higher in Asia compared to Europe or North America, the frequency of hereditary diffuse gastric cancer relative to the overall incidence of familial gastric carcinogenesis is lower in Asia [49]. In addition, due to several gene-association researches indicating potential differences among different geographical regions and ethnicities, consideration will be given to the possibility of variations in this association between Asians and Westerners [50–53]. However, several case–controls studies and a prospective study in Western populations also reported positive association between family history and gastric cancer [54–58]. Future research is warranted to further explore the possible differences in the association in different regions and race/ethnic groups.

Stronger association between gastric cancer incidence in more recent cohorts could be attributed to exposure to distinct environmental factors, such as unhealthy lifestyle habits or dietary patterns compared to older cohorts [37, 59], potentially elevating the risk of gastric cancer. Another possible factor is that recent cohorts may include more cases of gastric cancer that were previously undetected due to advancements in diagnosis and testing methods [60, 61], which may contribute to a higher risk of gastric cancer in the more recent cohorts.

We observed that individuals with family history of gastric cancer in either parents or siblings show a similarly comparable risk of developing gastric cancer, which is consistent with previous findings [31, 62]. The stronger association between gastric cancer and a family history involving parents or siblings may be attributed to genetic predisposition or shared exposure to common environmental factors, including carcinogenic *H. pylori* strains, shared dietary habits, or other factors that promote cancer development [54, 63]. Nevertheless, previous studies, primarily conducted on small scale case–control designs in Western populations, noted that the association tends to be more pronounced among siblings than parents [11, 32]. Our study findings indicate a significant association between male gastric cancer and family history involving brothers, father, and mother; whereas for female gastric cancer, a significant association is observed with maternal family history. Potential biological and hormonal factors may contribute to these sex-specific differences. Stronger gastric cancer association with sibling history could be attributed to the added influence of a family-shared environment in early ages, during which the dietary style usually forms and continues the rest of the life and also when it is a susceptible period for *H. pylori* attainment [64]. Our study found that the risk may be higher in females with mothers with gastric cancer and males with brothers with gastric cancer. While a previous cross-sectional study from South Korea [30] has examined the association between family history and gastric cancer risk by sex, our study provides additional insights in a prospective setting. Studies have suggested that daughters behave similarly to their mothers regarding eating behavior, physical activity, and BMI [65, 66]. Further research is warranted to elucidate the impact of a specific familial relationship between a history of gastric cancer and gastric cancer risk.

In our study, we found that gastric cancer patients with a family history of gastric cancer in Asia have higher mortality rates compared to those without a family history of gastric cancer. Although the impact of a family history of gastric cancer on the survival rates of gastric cancer patients remain uncertain, several previous studies suggest that family history may influence diagnosis stage and recurrence risk, particularly in advanced stages [67, 68]. Additionally, there is also a study suggesting that heightened awareness among those with a family history might lead to earlier diagnosis through increased screening, without necessarily improving survival outcomes [69]. Subsequent research endeavors should aim to explore the influence of screening behavior on individuals with a family history of gastric cancer and their subsequent mortality rates.

The major strengths of the current study include large-scale sample size of diverse Asian populations with long-term follow-up, the largest thus far in the investigation of family history of gastric cancer and gastric cancer risk, which allowed us the statistical power to perform several subgroup analyses, including the analysis of family relations. In addition, we had detailed individual-level data from each cohort for covariate adjustments and heterogeneity assessment, avoiding some of the constraints of meta-analysis based on pooled data from published studies. The number of exclusions owing to missing data on covariates was not particularly large (< 5%). All study participants were drawn from the general population, permitting our results to be extrapolated to the larger target population. Furthermore, gastric cancer diagnosis and mortality cases have been validated by National Cancer Registers in each country or by active long-term follow-up.

The current study has some limitations. First, family history of stomach cancer was self-reported, which may have resulted in misclassification. However, misclassification should not have occurred differentially between later cases and non-cases, therefore, it will not have resulted in biased association. Second, we lacked information about *H. pylori* infection. Although, *H. pylori* infections co-occur among family members [7], there is no evidence of heterogeneity of association between family history and gastric cancer risk among those with and without *H. pylori* infection [22, 70, 71]. Third, we assessed family history of gastric cancer and gastric cancer-related covariates at a single time point at baseline; yet the information on the family history of gastric cancer or the lifestyle of the participants’ may have changed during follow-up. Forth, despite the large sample size, only nine cohorts had information on anatomical subsites, and four cohorts with histological subtypes. Lastly, we did not have information on the specific number of gastric cancer relatives in the data. This may have allowed us to better quantify the genetic or environmental burden of the familial risk. Further studies need to explore a more detailed assessment of the number of gastric cancer relatives.

In this large consortium of prospective cohorts, we confirmed that having a family history of gastric cancer significantly increased the risk of gastric cancer in the Asian population. We were able to quantify the risk of gastric cancer for various risk factors, including sex and anatomical/histological subtypes. Those who have same-sex family members may have a stronger association with gastric cancer occurrence. Targeted education, screening, and intervention in high-risk groups may reduce the burden of gastric cancer.

### Supplementary Information

Below is the link to the electronic supplementary material.

Supplementary file1 (DOCX 81 KB)

## Data Availability

Access to the data is granted through the permission of the Asia Cohort Consortium. For more details, please visit https://www.asiacohort.org/about/workingwith/index.html. And send any inquiries to the Asia Cohort Consortium Coordinating Centre at the following email address: cc@asiacohort.org.

## References

[1] Sung H, Ferlay J, Siegel RL, Laversanne M, Soerjomataram I, Jemal A (2021). Global cancer statistics 2020: GLOBOCAN estimates of incidence and mortality worldwide for 36 cancers in 185 countries. CA Cancer J Clin.

[2] Morgan E, Arnold M, Camargo MC, Gini A, Kunzmann AT, Matsuda T (2022). The current and future incidence and mortality of gastric cancer in 185 countries, 2020–40: a population-based modelling study. EClinicalMedicine.

[3] Jun JK, Choi KS, Lee HY, Suh M, Park B, Song SH, et al. Effectiveness of the Korean National Cancer Screening Program in reducing gastric cancer mortality. Gastroenterology. 2017;152(6):1319–28.e7. 10.1053/j.gastro.2017.01.029 28147224

[4] Yashima K, Shabana M, Kurumi H, Kawaguchi K, Isomoto H (2022). Gastric cancer screening in Japan: a narrative review. J Clin Med.

[5] Januszewicz W, Turkot MH, Malfertheiner P, Regula J (2023). A global perspective on gastric cancer screening: which concepts are feasible, and when?. Cancers.

[6] Yaghoobi M, Bijarchi R, Narod SA (2009). Family history and the risk of gastric cancer. Br J Cancer.

[7] Luu MN, Quach DT, Hiyama T (2022). Screening and surveillance for gastric cancer: does family history play an important role in shaping our strategy?. Asia Pac J Clin Oncol.

[8] Drake I, Dias JA, Teleka S, Stocks T, Orho-Melander M (2020). Lifestyle and cancer incidence and mortality risk depending on family history of cancer in two prospective cohorts. Int J Cancer.

[9] Nam JH, Choi IJ, Cho SJ, Kim CG, Lee JY, Nam SY (2011). *Helicobacter pylori* infection and histological changes in siblings of young gastric cancer patients. J Gastroenterol Hepatol.

[10] Man J, Ni Y, Yang X, Zhang T, Yuan Z, Chen H (2021). Healthy lifestyle factors, cancer family history, and gastric cancer risk: a population-based case-control study in China. Front Nutr.

[11] Vitelli-Storelli F, Rubin-Garcia M, Pelucchi C, Benavente Y, Bonzi R, Rota M (2021). Family history and gastric cancer risk: a pooled investigation in the Stomach Cancer Pooling (STOP) Project Consortium. Cancers (Basel).

[12] Yusefi AR, Bagheri Lankarani K, Bastani P, Radinmanesh M, Kavosi Z (2018). Risk factors for gastric cancer: a systematic review. Asian Pac J Cancer Prev.

[13] Zhang R, Li H, Li N, Shi JF, Li J, Chen HD (2021). Risk factors for gastric cancer: a large-scale, population-based case-control study. Chin Med J (Engl).

[14] Song M, Rolland B, Potter JD, Kang D (2012). Asia Cohort Consortium: challenges for collaborative research. J Epidemiol.

[15] Rolland B, Smith BR, Potter JD (2011). Coordinating centers in cancer epidemiology research: the Asia Cohort Consortium coordinating center. Cancer Epidemiol Biomarkers Prev.

[16] TAC C. https://www.asiacohort.org/index.html.

[17] Anderson WF, Rabkin CS, Turner N, Fraumeni JF, Rosenberg PS, Camargo MC (2018). The changing face of noncardia gastric cancer incidence among US non-Hispanic whites. J Natl Cancer Inst.

[18] Bakir T, Can G, Siviloglu C, Erkul S (2003). Gastric cancer and other organ cancer history in the parents of patients with gastric cancer. Eur J Cancer Prev.

[19] Setia N, Clark JW, Duda DG, Hong TS, Kwak EL, Mullen JT (2015). Familial gastric cancers. Oncologist.

[20] Boland CR, Yurgelun MB (2017). Historical perspective on familial gastric cancer. Cell Mol Gastroenterol Hepatol.

[21] Lott PC, Carvajal-Carmona LG (2018). Resolving gastric cancer aetiology: an update in genetic predisposition. Lancet Gastroenterol Hepatol.

[22] Choi YJ, Kim N (2016). Gastric cancer and family history. Korean J Intern Med.

[23] Decourtye-Espiard L, Guilford P (2023). Hereditary diffuse gastric cancer. Gastroenterology.

[24] Usui Y, Taniyama Y, Endo M, Koyanagi YN, Kasugai Y, Oze I (2023). *Helicobacter pylori*, homologous-recombination genes, and gastric cancer. N Engl J Med.

[25] Rokkas T, Sechopoulos P, Pistiolas D, Margantinis G, Koukoulis G (2010). *Helicobacter pylori* infection and gastric histology in first-degree relatives of gastric cancer patients: a meta-analysis. Eur J Gastroenterol Hepatol.

[26] Choi IJ, Kim CG, Lee JY, Kim YI, Kook MC, Park B (2020). Family history of gastric cancer and *Helicobacter pylori* treatment. N Engl J Med.

[27] Koyama T, Yoshiike N (2019). Association between parent and child dietary sodium and potassium intakes: Aomori Prefectural Health and Nutrition Survey, 2016. Nutrients.

[28] Cheng XJ, Lin JC, Tu SP (2016). Etiology and prevention of gastric cancer. Gastrointest Tumors.

[29] Morais S, Costa A, Albuquerque G, Araújo N, Pelucchi C, Rabkin CS (2022). Salt intake and gastric cancer: a pooled analysis within the Stomach cancer Pooling (StoP) Project. Cancer Causes Control.

[30] Choi HG, Chun W, Jung KH (2022). Association between gastric cancer and the family history of gastric cancer: a cross-sectional study using Korean Genome and Epidemiology Study data. Eur J Cancer Prev.

[31] Jung YS, Xuan Tran MT, Park B, Moon CM (2022). Association between family history of gastric cancer and the risk of gastric cancer and adenoma: a nationwide population-based study. Am J Gastroenterol.

[32] Song M, Camargo MC, Weinstein SJ, Best AF, Mannisto S, Albanes D (2018). Family history of cancer in first-degree relatives and risk of gastric cancer and its precursors in a Western population. Gastric Cancer.

[33] de Martel C, Georges D, Bray F, Ferlay J, Clifford GM. Global burden of cancer attributable to infections in 2018: a worldwide incidence analysis. Lancet Glob Health. 2020;8(2):e180–90. 10.1016/S2214-109X(19)30488-7 31862245

[34] Kamangar F, Dawsey SM, Blaser MJ, Perez-Perez GI, Pietinen P, Newschaffer CJ (2006). Opposing risks of gastric cardia and noncardia gastric adenocarcinomas associated with *Helicobacter pylori* seropositivity. J Natl Cancer Inst.

[35] Hu N, Wang Z, Song X, Wei L, Kim BS, Freedman ND (2016). Genome-wide association study of gastric adenocarcinoma in Asia: a comparison of associations between cardia and non-cardia tumours. Gut.

[36] Lou L, Wang L, Zhang Y, Chen G, Lin L, Jin X (2020). Sex difference in incidence of gastric cancer: an international comparative study based on the Global Burden of Disease Study 2017. BMJ Open.

[37] Nagase H, Ogino K, Yoshida I, Matsuda H, Yoshida M, Nakamura H (1996). Family history-related risk of gastric cancer in Japan: a hospital-based case-control study. Jpn J Cancer Res.

[38] Palli D, Galli M, Caporaso NE, Cipriani F, Decarli A, Saieva C (1994). Family history and risk of stomach cancer in Italy. Cancer Epidemiol Biomarkers Prev.

[39] Shin CM, Kim N, Yang HJ, Cho SI, Lee HS, Kim JS (2010). Stomach cancer risk in gastric cancer relatives: interaction between *Helicobacter pylori* infection and family history of gastric cancer for the risk of stomach cancer. J Clin Gastroenterol.

[40] Petrovchich I, Ford JM (2016). Genetic predisposition to gastric cancer. Semin Oncol.

[41] Zhou XF, He YL, Song W, Peng JJ, Zhang CH, Li W (2009). Comparison of patients by family history with gastric and non-gastric cancer. World J Gastroenterol.

[42] Kharazmi E, Fallah M, Sundquist K, Hemminki K (2012). Familial risk of early and late onset cancer: nationwide prospective cohort study. BMJ.

[43] Archambault AN, Su YR, Jeon J, Thomas M, Lin Y, Conti DV, et al. Cumulative burden of colorectal cancer-associated genetic variants is more strongly associated with early-onset vs late-onset cancer. Gastroenterology. 2020;158(5):1274–86.e12. 10.1053/j.gastro.2019.12.012 PMC7103489 31866242

[44] Kluijt I, Sijmons RH, Hoogerbrugge N, Plukker JT, de Jong D, van Krieken JH (2012). Familial gastric cancer: guidelines for diagnosis, treatment and periodic surveillance. Fam Cancer.

[45] Yaghoobi M, Rakhshani N, Sadr F, Bijarchi R, Joshaghani Y, Mohammadkhani A (2004). Hereditary risk factors for the development of gastric cancer in younger patients. BMC Gastroenterol.

[46] Takeshima H, Ushijima T (2019). Accumulation of genetic and epigenetic alterations in normal cells and cancer risk. NPJ Precis Oncol.

[47] Madhavan S, Bullis E, Myers R, Zhou CJ, Cai EM, Sharma A (2019). Awareness of family health history in a predominantly young adult population. PLoS ONE.

[48] Teo CH, Ng CJ, White A (2017). Factors influencing young men’s decision to undergo health screening in Malaysia: a qualitative study. BMJ Open.

[49] Corso GMD, Corso G, Roviello F (2013). Frequency of familial gastric cancer. Spotlight on familial and hereditary gastric cancer.

[50] Jin G, Wang L, Chen W, Hu Z, Zhou Y, Tan Y, Wang J (2007). Variant alleles of TGFB1 and TGFBR2 are associated with a decreased risk of gastric cancer in a Chinese population. Int J Cancer.

[51] Kamangar F, Cheng C, Abnet CC, Rabkin CS (2006). Interleukin-1B polymorphisms and gastric cancer risk—a meta-analysis. Cancer Epidemiol Biomarkers Prev.

[52] Kim N, Cho S, Yim J-Y, Kim JM, Lee DH, Park JH, Kim JS (2006). The effects of genetic polymorphisms of IL-1 and TNF-A on *Helicobacter pylori*-induced gastroduodenal diseases in Korea. Helicobacter.

[53] Savage SA, Abnet CC, Mark SD, Qiao Y-L, Dong Z-W, Dawsey SM, Taylor PR (2004). Variants of the IL8 and IL8RB genes and risk for gastric cardia adenocarcinoma and esophageal squamous cell carcinoma. Cancer Epidemiol Biomarkers Prev.

[54] Song H, Ekheden IG, Ploner A, Ericsson J, Nyren O, Ye W (2018). Family history of gastric mucosal abnormality and the risk of gastric cancer: a population-based observational study. Int J Epidemiol.

[55] Lissowska J, Groves FD, Sobin LH, Fraumeni JF, Nasierowska-Guttmejer A, Radziszewski J (1999). Family history and risk of stomach cancer in Warsaw, Poland. Eur J Cancer Prev.

[56] Brenner H, Arndt V, Stürmer T, Stegmaier C, Ziegler H, Dhom G (2000). Individual and joint contribution of family history and *Helicobacter pylori* infection to the risk of gastric carcinoma. Cancer.

[57] Dhillon PK, Farrow DC, Vaughan TL, Chow WH, Risch HA, Gammon MD (2001). Family history of cancer and risk of esophageal and gastric cancers in the United States. Int J Cancer.

[58] Jiang X, Tseng CC, Bernstein L, Wu AH (2014). Family history of cancer and gastroesophageal disorders and risk of esophageal and gastric adenocarcinomas: a case-control study. BMC Cancer.

[59] Shim JS, Shim SY, Cha HJ, Kim J, Kim HC (2021). Socioeconomic characteristics and trends in the consumption of ultra-processed foods in Korea from 2010 to 2018. Nutrients.

[60] Ryu JE, Choi E, Lee K, Jun JK, Suh M, Jung KW (2022). Trends in the performance of the Korean National Cancer Screening Program for gastric cancer from 2007 to 2016. Cancer Res Treat.

[61] Mabe K, Inoue K, Kamada T, Kato K, Kato M, Haruma K (2022). Endoscopic screening for gastric cancer in Japan: current status and future perspectives. Dig Endosc.

[62] Foschi R, Lucenteforte E, Bosetti C, Bertuccio P, Tavani A, La Vecchia C (2008). Family history of cancer and stomach cancer risk. Int J Cancer.

[63] Queiroz DM, Silva CI, Goncalves MH, Braga-Neto MB, Fialho AB, Fialho AM (2012). Higher frequency of cagA EPIYA-C phosphorylation sites in *H. pylori* strains from first-degree relatives of gastric cancer patients. BMC Gastroenterol.

[64] Weyermann M, Rothenbacher D, Brenner H (2009). Acquisition of *Helicobacter pylori* infection in early childhood: independent contributions of infected mothers, fathers, and siblings. Am J Gastroenterol.

[65] Shaban LH, Vaccaro JA, Sukhram SD, Huffman FG (2018). Do mothers affect daughter’s behaviors? Diet, physical activity, and sedentary behaviors in Kuwaiti mother-daughter dyads. Ecol Food Nutr.

[66] Dhana K, Haines J, Liu G, Zhang C, Wang X, Field AE (2018). Association between maternal adherence to healthy lifestyle practices and risk of obesity in offspring: results from two prospective cohort studies of mother-child pairs in the United States. BMJ.

[67] Kim HJ, Kwon M, Kim N, Lee JB, Won S (2020). The influence of family history on stage and survival of gastric cancer according to the TGFB1 C-509T polymorphism in Korea. Gut Liver.

[68] Han MA, Oh MG, Choi IJ, Park SR, Ryu KW, Nam BH (2012). Association of family history with cancer recurrence and survival in patients with gastric cancer. J Clin Oncol.

[69] Schoenfeld P (2018). Evidence-based guidelines for screening individuals with a family history of colorectal cancer-more questions than answers. Gastroenterology.

[70] Lee J, Chung SJ, Choi JM, Han YM, Kim JS (2020). Clinicopathologic characteristics and long-term outcome of gastric cancer patients with family history: seven-year follow-up study for Korean health check-up subjects. Gastroenterol Res Pract.

[71] Lim SH, Kwon JW, Kim N, Kim GH, Kang JM, Park MJ (2013). Prevalence and risk factors of *Helicobacter pylori* infection in Korea: nationwide multicenter study over 13 years. BMC Gastroenterol.

