# Supplementary material for: Family history and gastric cancer incidence and mortality in Asia: a pooled analysis of more than half a million participants
Source: Gastric Cancer. 2024 Apr 22;27(4):701–13. doi: 10.1007/s10120-024-01499-1 (PMC11193690; doi:10.1007/s10120-024-01499-1)
Supplement: Supplementary file 1 — Supplementary file1 (DOCX 81 KB) [file 10120_2024_1499_MOESM1_ESM.docx]

**Supplementary Materials**

[Supplementary Table 1. Characteristics of Cohorts in the Current Study of Asia Cohort Consortium 2](#_Toc154755120)

[Supplementary Table 2. Exclusion Criteria for the Selected Participants of Gastric Cancer Incidence Analysis by Each Cohort 5](#_Toc154755121)

[Supplementary Table 3. Exclusion Criteria for the Selected Participants of Gastric Cancer Mortality Analysis by Each Cohort 6](#_Toc154755122)

[Supplementary Table 4. Baseline Characteristics of Cohort Studies in the Pooled Mortality Analysis (N=531,137) 7](#_Toc154755123)

[Supplementary Table 5. Association Between Family History of Gastric Cancer and Gastric Cancer Mortality 8](#_Toc154755124)

[Supplementary Table 6. Association Between Family History of Gastric Cancer and Gastric Cancer Mortality by the Subgroups 9](#_Toc154755125)

[Supplementary Table 7. Association Between Family History of Gastric Cancer and Gastric Cancer Mortality by Gastric Cancer Subtypes 10](#_Toc154755126)

[Supplementary Table 8. Association Between Family History of Gastric Cancer and Gastric Cancer Mortality Grouping According to the Relation of Family History 11](#_Toc154755127)

[Supplementary Reference 1](#_Toc154755127)2

Supplementary Table 1. Characteristics of Cohorts in the Current Study of Asia Cohort Consortium

| Cohort Name | Study Location, Total Participant Number, and Characteristics | Family History of Gastric Cancer Assessment | Outcome Assessment | Gastric Cancer-Related Information | Covariates Included in Models |
| --- | --- | --- | --- | --- | --- |
| 3 Prefecture Miyagi1 | N= 100,610 aged over 40 rsidents of Miyagi, Aichi, and Osaka | Self-administered | Vital status and date of death were collected from residence certificates by the local government, and cause of death was identified using vital statistics. Cancer incidence and the date of diagnosis were collected from local cancer registry data. | Anatomical subsite (ICD-9): Cardia (151.0), Non-cardia (151.1, 151.2, 151.3, 151.4, 151.5, 151.6, 151.7, 151.8) | Sex, smoking (ever/never), alcohol consumption (ever/never), and body mass index (continuous) |
| 3 Prefecture Aichi1 | N=33538 aged over 40 residents of Nagoya and Inuyama City of Aichi prefecture, Japan | Self-administered | Death and cancer incidence information were retrieved from residence certificates, vital statistics, and the local cancer registry· | Anatomical subsite (ICD-10): Cardia (C160), Non-cardia (C161,C162, C163, C164, C165, C166)  Histological subtype: intestinal type (8012, 8021, 8022, 8031, 8032, 8046,8050, 8082, 8143, 8144, 8201, 8210, 8211, 8220, 8221, 8255, 8260, 8261, 8262, 8263, 8310, 8323, 8480, 8481, 8510, 8512, 8570, 8576), diffuse type (8020, 8041, 8044, 8141, 8142, 8145, 8490, 8806) | Sex, smoking (ever/never), alcohol consumption (ever/never), and body mass index (continuous) |
| Japan Collaborative Cohort Study for Evaluation of Cancer Risk (JACC Study) 2 | N=110585 (46395 men and 64190 women) aged 40 to 79 years residents in Hokkaido, Tohoku, Kanto, Chubu, Kinkim Chugoku, and Kyushu districts, Japan | Self-administered | Cancer incidence and causes of death were collected through population-based registers or local hospital records | Anatomical subsite (ICD-10): Cardia (C160), Non-cardia (C161,C162, C163, C164, C165, C166)  histological subtype: intestinal type (8012, 8021, 8022, 8031, 8032, 8046,8050, 8082, 8143, 8144, 8201, 8210, 8211, 8220, 8221, 8255, 8260, 8261, 8262, 8263, 8310, 8323, 8480, 8481, 8510, 8512, 8570, 8576), diffuse type (8020, 8041, 8044, 8141, 8142, 8145, 8490, 8806) | Sex, smoking (ever/never), alcohol consumption (ever/never), education level (low/high), and body mass index (continuous) |
| Miyagi Cohort 3 | N=51,921 aged 40 to 64 years residents in 14 municipalities of Miyagi Prefecure, Japan | Self-administered | Linkage of these baseline data with the cancer incidence data of a population-based cancer registry | Anatomical subsite (ICD-9): Cardia (151.0), Non-cardia (151.1, 151.2, 151.3, 151.4, 151.5, 151.6, 151.7, 151.8) | Sex, smoking (ever/never), alcohol consumption (ever/never), education level (low/high), body mass index (continuous) |
| Japan Public Health Center-Based Prospective Study I (JPHC1) 4 | N=61595 aged 40 to 59 residents of Iwate, Akita, Tokyo, Nagano, and Okinawa, Japan | Self-administered | Participants in the study were followed up for the occurrence of cancer and death using data from cancer and residential registries | Anatomical subsite (ICD-10): Cardia (C160), Non-cardia (C161,C162, C163, C164, C165, C166)  Histological subtype: intestinal type (8012, 8021, 8022, 8031, 8032, 8046,8050, 8082, 8143, 8144, 8201, 8210, 8211, 8220, 8221, 8255, 8260, 8261, 8262, 8263, 8310, 8323, 8480, 8481, 8510, 8512, 8570, 8576), diffuse type (8020, 8041, 8044, 8141, 8142, 8145, 8490, 8806) | Sex, smoking (ever/never), alcohol consumption (ever/never), education level (low/high), and body mass index (continuous) |
| Japan Public Health Center-Based Prospective Study II (JPHC2) 4 | N=78825 aged 40 to 69 residents of Ibaraki, Niigata, Osaka, Kochi, Nagasaki, and Okinawa, Japan | Self-administered | Same as JPHC1 | Anatomical subsite (ICD-10): Cardia (C160), Non-cardia (C161,C162, C163, C164, C165, C166)  Histological subtype: intestinal type (8012, 8021, 8022, 8031, 8032, 8046,8050, 8082, 8143, 8144, 8201, 8210, 8211, 8220, 8221, 8255, 8260, 8261, 8262, 8263, 8310, 8323, 8480, 8481, 8510, 8512, 8570, 8576), diffuse type (8020, 8041, 8044, 8141, 8142, 8145, 8490, 8806) | Sex, smoking (ever/never), alcohol consumption (ever/never), and body mass index (continuous) |
| Ohsaki National Health Insurance Cohort Study3 | N=54,996 aged 40 to 79 years living in the catchment area of Ohsaki Public Health Center, Miyagi, Japan. | Self-administered | Same as Miyagi | Anatomical subsite (ICD-10): Cardia (C160), Non-cardia (C161,C162, C163, C164, C165, C166) | Sex, smoking (ever/never), alcohol consumption (ever/never), education level (low/high), body mass index (continuous) |
| Linxian General Population Trial Cohort5 | N-=29,584, residents 40–69 years of age with no history of cancer or debilitating disease from four northern com- munes in Linxian, a rural county in Henan Province | Self-administered | For new cancer diagnoses and deaths, diagnostic materi- als were collected and cancer diagnoses were verified by the panel of American and Chinese experts (1991 to 1996) or senior Chinese diagnosticians from Beijing (1996–2001). |  | Sex, smoking (ever/never), alcohol consumption (ever/never), education level (low/high), body mass index (continuous) |
| Shanghai Men’s Health Study (SMHS)6 | N=61480 cancer-free men aged 40 to 74 in urban Shanghai communities, China | Self-administered | Record linkages to the Shanghai Cancer Registry (SCR), Shanghai Vital Statistics Registry, the Shanghai Residential Registry, and the Changning District Health Information System |  | Smoking (ever/never), alcohol consumption (ever/never), education level (low/high), body mass index (continuous) |
| Shanghai Women’s Health Study (SWHS)7 | N=74942 women aged 40 to 70 years in urban Shanghai communities, China | Self-administered | Study participants were followed by an in-person assessment every two years and were routinely linked to cancer and vital statistic registries |  | Smoking (ever/never), alcohol consumption (ever/never), education level (low/high), body mass index (continuous) |
| Korea Multicenter Cancer Cohort Study (KMCC)8 | N=19688 cancer-free women and men over 18 years old residents in Haman, Choongju, Uljin, and Pohang, Republic of Korea | Interviewer-administered | Cancer incidence and cause of death information were retrieved from the central and regional cancer registries, health insurance medical records databases, and national death certificates | Anatomical subsite (ICD-10): Cardia (C160), Non-cardia (C161, C162, C163, C164, C165, C166) | Sex, smoking (ever/never), alcohol consumption (ever/never), education level (low/high), and body mass index (continuous) |
| Korea National Cancer Center Cohort Study (KNCC)9 | N=16304 men and women over 30 years of age, residents in Haman, Sancheong, Changwon, Chungju, Chuncheon, Republic of Korea | Interviewer-administered | Information on cancer incidence and cause of death was retrieved annually through the Korea National Cancer Incidence Database of the Korean Central Cancer Registry and the Cause of Death Database of Statistics | Anatomical subsite (ICD-10): Cardia (C160), Non-cardia (C161, C162, C163, C164, C165, C166) | Sex, smoking (ever/never), alcohol consumption (ever/never), education level (low/high), and body mass index (continuous) |

Supplementary Table 2. Exclusion Criteria for the Selected Participants of Gastric Cancer Incidence Analysis by Each Cohort

| Country | Cohort Name | Period of Enrollment, Year | Participants, N | Exclusion Criteria^a^ | | | | | Total Excluded Participants | Participants Included in the Final Analysis |
| --- | --- | --- | --- | --- | --- | --- | --- | --- | --- | --- |
|  |  |  |  | No Information on Age or Sex | Inaccurate Follow-up Information | No Information on Gastric Cancer | No Information on Family History of Gastric Cancer | Gastric Cancer Diagnosed Before Enroll |  |  |
| Japan | Miyagi3P | 1984 | 30,809 | 0 | 1 | 0 | 0 | 535 | 536 | 30,809 |
|  | Aichi3P | 1985 | 24,917 | 0 | 33 | 0 | 8,547 | 74 | 8,612 | 24,917 |
|  | JACC | 1988-1990 | 81,494 | 0 | 0 | 0 | 3,880 | 1,163 | 5,011 | 81,494 |
|  | Miyagi | 1990 | 46,132 | 0 | 1 | 0 | 925 | 561 | 1,473 | 46,132 |
|  | JPHC | 1990-1992 | 42,136 | 0 | 45 | 0 | 0 | 916 | 960 | 42,136 |
|  | JPHC2 | 1992-1995 | 55,317 | 0 | 37 | 2 | 0 | 1,218 | 1,255 | 55,317 |
|  | Ohsaki | 1995 | 49,498 | 1 | 4 | 1 | 0 | 1,752 | 1,755 | 49,498 |
| China | Linxian | 1984-1987 | 29,458 | 0 | 11 | 0 | 125 | 0 | 136 | 29,458 |
|  | SMHS | 2001-2006 | 61,464 | 0 | 5 | 0 | 0 | 0 | 5 | 61,464 |
|  | SWHS | 1996-2000 | 73,355 | 0 | 9 | 0 | 0 | 1,576 | 1,585 | 73,355 |
| South Korea | SeoulM | 1992-1993 | 14,533 | 525 | 0 | 14,533 | 14,533 | 0 | 14,533 | 0 |
|  | KMCC | 1993-2005 | 20,323 | 5 | 96 | 5 | 0 | 260 | 313 | 20,323 |
|  | KNCC | 2007-2015 | 35,605 | 0 | 2,961 | 3,073 | 2,063 | 2,122 | 7,146 | 35,605 |
| Bangladesh | HEALS | 2000-2002 | 35,051 | 1 | 15,018 | 33,620 | 35,051 | 3 | 35,051 | 0 |
|  | Total |  | 628,879 | 532 | 18,221 | 51,234 | 65,124 | 10,180 | 78,371 | 550,508 |
| Abbreviation: Miyagi3p, Three Prefecture Cohort Study Miyagi; Aichi3p, Three Prefecture Cohort Study Aichi; JACC, Japan Collaborative Cohort Study; Miyagi, Miyagi Cohort Study; JPHC, Japan Public Health Center-based prospective Study; Ohsaki, Ohsaki National Health Insurance Cohort Study; Linxian, Linxian General population Trial Cohort; SWHS, Shanghai Women’s Health Study; SMHS, Shanghai Men’s Health Study; SeoulM, Seoul Male Cancer Cohort; KMCC, Korean Multi-center Cancer Cohort Study; KNCC, Korean National Cancer Center Cohort; HEALS, Health Effects for Arsenic Longitudinal Study Bangladesh; N, number. | | | | | | | | | | |
| ^a^ Number of participants in each exclusion criteria are not exclusive | | | | | | | | | | |

| Country | Cohort  Name | Period of Enrollment, Year | Participants,  N | Exclusion Criteria^a^ | | | Total Excluded Participants | Participants Included in the Final Analysis |
| --- | --- | --- | --- | --- | --- | --- | --- | --- |
|  |  |  |  | No Information on Age and Sex | Inaccurate Follow-up Information | No Information on Family History of Gastric Cancer |  |  |
| Japan | Miyagi3P | 1984 | 31,345 | 0 | 0 | 0 | 0 | 31,345 |
|  | Aichi3P | 1985 | 33,529 | 0 | 19 | 8,547 | 8,557 | 24,972 |
|  | JACC | 1988-1990 | 86,505 | 0 | 0 | 3,880 | 3,880 | 82,625 |
|  | Miyagi | 1990 | 47,605 | 0 | 1 | 925 | 926 | 46,679 |
|  | JPHC | 1990-1992 | 43,096 | 0 | 45 | 0 | 45 | 43,051 |
|  | JPHC2 | 1992-1995 | 56,572 | 0 | 37 | 0 | 37 | 56,535 |
|  | Ohsaki | 1995 | 51,253 | 1 | 4 | 0 | 4 | 51,249 |
| China | Linxian | 1984-1987 | 29,594 | 0 | 7 | 29,463 | 29,463 | 0 |
|  | SMHS | 2001-2006 | 74,940 | 0 | 3 | 0 | 3 | 74,937 |
|  | SWHS | 1996-2000 | 61,469 | 0 | 5 | 0 | 5 | 61,464 |
| South Korea | SeoulM | 1992-1993 | 14,533 | 525 | 0 | 14,533 | 14,533 | 0 |
|  | KMCC | 1993-2005 | 20,636 | 5 | 5 | 0 | 5 | 20,631 |
|  | KNCC | 2007-2015 | 42,751 | 0 | 3,073 | 2,063 | 5,102 | 37,649 |
| Bangladesh | HEALS | 2000-2002 | 35,051 | 1 | 15,018 | 35,051 | 35,051 | 0 |
|  | Total |  | 628,879 | 532 | 18,221 | 65,124 | 78,371 | 531,137 |
| Abbreviation: Miyagi3p, Three Prefecture Cohort Study Miyagi; Aichi3p, Three Prefecture Cohort Study Aichi; JACC, Japan Collaborative Cohort Study; Miyagi, Miyagi Cohort Study; JPHC, Japan Public Health Center-based prospective Study; Ohsaki, Ohsaki National Health Insurance Cohort Study; Linxian, Linxian General population Trial Cohort; SWHS, Shanghai Women’s Health Study; SMHS, Shanghai Men’s Health Study; SeoulM, Seoul Male Cancer Cohort; KMCC, Korean Multi-center Cancer Cohort Study; KNCC, Korean National Cancer Center Cohort; HEALS, Health Effects for Arsenic Longitudinal Study Bangladesh; N, number. | | | | | | | | |
| ^a^ The numbers of participants in each exclusion criteria are not exclusive | | | | | | | | |

Supplementary Table 3. Exclusion Criteria for the Selected Participants of Gastric Cancer Mortality Analysis by Each Cohort 

Supplementary Table 4. Baseline Characteristics of Cohort Studies in the Pooled Mortality Analysis (N=531,137)

| Cohort Name | Participants, N | Period of Enrollment, Year | Years of  Follow-up,  Mean (SD) | Age at Enrollment, Mean (SD) | Body Mass Index,  kg/m^2^, Mean (SD) | Male,  % | Ever Smoking,  % | Ever Drinking,  % | Low Education^a^,  % | Family History of Gastric Cancer, % | Death of Gastric Cancer,  N |
| --- | --- | --- | --- | --- | --- | --- | --- | --- | --- | --- | --- |
| Japan |  |  |  |  |  |  |  |  |  |  |  |
| Miyagi3P | 31,345 | 1984 | 7.6 (2.6) | 57.3 (11.3) | 23.3 (3.4) | 44.6 | 30.6 | 47.2 | NA | 11.7 | 287 |
| Aichi3P | 24,972 | 1985 | 11.6 (5.1) | 55.4 (10.9) | 22.1 (3.0) | 47.6 | 47.4 | 61.4 | NA | 13.4 | 275 |
| JACC | 82,625 | 1988-1990 | 16.1 (5.7) | 57.6 (10.0) | 22.8 (3.6) | 41.9 | 35.5 | 46.6 | 17.8 | 11.3 | 1,149 |
| Miyagi | 46,679 | 1990 | 21.5 (6.0) | 52.1 (7.5) | 23.6 (3.0) | 47.9 | 42.2 | 52.0 | 54.8 | 10.7 | 802 |
| JPHC | 43,051 | 1990-1992 | 21.0 (4.3) | 49.6 (5.9) | 23.6 (3.0) | 47.9 | 40.1 | 49.8 | 49.8 | 6.9 | 419 |
| JPHC2 | 56,535 | 1992-1995 | 17.7(4.2) | 54.3 (8.8) | 23.5 (3.1) | 47.4 | 39.8 | 48.8 | NA | 5.9 | 654 |
| Ohsaki | 51,249 | 1995 | 10.7 (4.3) | 60.5 (10.3) | 23.6 (3.4) | 48.0 | 41.2 | 49.6 | 56.1 | 13.6 | 585 |
| China |  |  |  |  |  |  |  |  |  |  |  |
| SWHS | 74,937 | 1996-2000 | 17.4 (3.0) | 52.6 (9.1) | 24.0 (3.4) | 0 | 2.8 | 2.2 | 21.6 | 5.9 | 401 |
| SMHS | 61,464 | 2001-2006 | 11.9 (2.4) | 55.4 (9.7) | 23.7 (3.1) | 100 | 69.6 | 33.7 | 6.6 | 6.4 | 410 |
| South Korea |  |  |  |  |  |  |  |  |  |  |  |
| KMCC | 20,631 | 1993-2005 | 13.9 (4.7) | 54.1 (14.3) | 23.6 (3.3) | 39.9 | 36.2 | 41.0 | 72.6 | 4.9 | 192 |
| KNCC | 37,649 | 2007-2015 | 9.3 (3.3) | 49.9 (9.3) | 23.8 (3.0) | 50.7 | 44.3 | 66.3 | 7.0 | 17.5 | 20 |
| Total (12) | 531,137 |  | 15.3 (5.9) | 54.6 (10.1) | 23.5 (3.3) | 45.9 | 55.8 | 52.7 | 21.4 | 9.2 | 5,194 |
| Abbreviation: N, number; SD, standard deviation; NA, not available | | | | | | | | | | | |
| Miyagi3p, Three Prefecture Cohort Study Miyagi; Aichi3p, Three Prefecture Cohort Study Aichi; JACC, Japan Collaborative Cohort Study; Miyagi, Miyagi Cohort Study; JPHC, Japan Public Health Center-based prospective Study; Ohsaki, Ohsaki National Health Insurance Cohort Study; SWHS, Shanghai Women’s Health Study; SMHS, Shanghai Men’s Health Study; KMCC, Korean Multi-center Cancer Cohort Study; KNCC, Korean National Cancer Center Cohort. | | | | | | | | | | | |
| ^a^ Primary school education or below | | | | | | | | | | | |

| Country | Cohort Name | Baseline Survey Years | Participants, N | Death of Gastric Cancer, N | Family History of Gastric Cancer among Cases, % |  |  | HR^a^ (95% CI) | Weight  % |
| --- | --- | --- | --- | --- | --- | --- | --- | --- | --- |
| Gastric Cancer Mortality | | |  |  |  |  |  |  |  |
| Japan | Miyagi3P | 1984 | 31,345 | 287 | 14.3 |  |  | 1.25 (0.90-1.74) | 6.35 |
|  | Aichi3P | 1985 | 24,972 | 275 | 20.0 |  |  | 1.56 (1.16-2.09) | 7.93 |
|  | JACC | 1988-1990 | 82,625 | 1,149 | 14.8 |  |  | 1.32 (1.12-1.55 | 25.99 |
|  | Miyagi | 1990 | 46,679 | 802 | 13.7 |  |  | 1.23 (1.01-1.51) | 17.12 |
|  | JPHC | 1990-1992 | 43,051 | 419 | 9.8 |  |  | 1.36 (0.98-1.88) | 6.68 |
|  | JPHC2 | 1992-1995 | 56,535 | 654 | 7.8 |  |  | 1.34 (1.01-1.78) | 8.52 |
|  | Ohsaki | 1995 | 51,249 | 585 | 16.8 |  |  | 1.25 (1.01-1.55) | 14.79 |
| China | SWHS | 1996-2000 | 74,937 | 401 | 8.0 |  |  | 1.36 (0.95-1.96) | 5.34 |
|  | SMHS | 2001-2006 | 61,464 | 410 | 8.8 |  |  | 1.38 (0.98-1.94) | 5.94 |
| South Korea | KMCC | 1993-2005 | 20,631 | 192 | 2.6 |  |  | 0.65 (0.27-1.58) | 0.88 |
|  | KNCC | 2007-2015 | 37,464 | 20 | 15.0 |  |  | 1.06 (0.31-3.63) | 0.46 |
|  | Overall (I-squared=0.0%, p=0.916) | | | |  |  |  | 1.31 (1.20-1.42) | 100 |
|  | | | | | | | | | |
|  | | | | | | | | | |
| Abbreviation: Miyagi3p, Three Prefecture Cohort Study Miyagi; Aichi3p, Three Prefecture Cohort Study Aichi; JACC, Japan Collaborative Cohort Study; Miyagi, Miyagi Cohort Study; JPHC, Japan Public Health Center-based prospective Study; Ohsaki, Ohsaki National Health Insurance Cohort Study; SWHS, Shanghai Women’s Health Study; SMHS, Shanghai Men’s Health Study; KMCC, Korean Multi-center Cancer Cohort Study; KNCC, Korean National Cancer Center Cohort; N, number; HR, hazard ratio. | | | | | | | | | |
| ^a^ HR refers to a summary estimate of effects based on a random-effects model, adjusted for by sex, smoking (ever/never), alcohol consumption (ever/never), and education (low/high) and body mass index (kg/m2, continuous) depending on availability in each cohort | | | | | | | | | |

Supplementary Table 5. Association Between Family History of Gastric Cancer and Gastric Cancer Mortality

Supplementary Table 6. Association Between Family History of Gastric Cancer and Gastric Cancer Mortality by the Subgroups

| Subgroup | Studies, N | Participants, N | Death of Castric Cancer, N | Family History of Gastric Cancer among Cases, % | HR (95%CI)^a^ | Heterogeneity  within Subgroups^b^ | | Heterogeneity between Subgroups^c^ |
| --- | --- | --- | --- | --- | --- | --- | --- | --- |
|  |  |  |  |  |  | I^2^ value (%) | *p*-value |  |
| Country | | | | | | | | 0.34 |
| Japan | 7 | 336,456 | 4,171 | 13.6 | 1.31 (1.20-1.43) | 0 | 0.92 |  |
| China | 2 | 136,401 | 811 | 8.4 | 1.37 (1.07-1.76) | 0 | 0.96 |  |
| South Korea | 2 | 58,280 | 212 | 3.8 | 0.77 (0.37-1.58) | 0 | 0.53 |  |
| Sex | | | | | | | | 0.32 |
| Male | 10 | 243,678 | 3,396 | 11.8 | 1.32 (1.19-1.47) | 0 | 0.45 |  |
| Female | 10 | 287,459 | 1,798 | 13.4 | 1.45 (1.27-1.67) | 0 | 0.78 |  |
| Enrollment years | | | | | | | | 0.45 |
| 1980-1989 | 3 | 138,942 | 1,711 | 15.5 | 1.33 (1.17-1.52) | 0 | 0.51 |  |
| 1990-1999 | 5 | 272,451 | 2,861 | 11.6 | 1.28 (1.14-1.44) | 0 | 0.97 |  |
| After 2000 | 3 | 119,744 | 622 | 7.1 | 1.35 (0.97-1.88) | 18.7 | 0.29 |  |
| Age at enrollment, years | | | | | | | | 0.30 |
| <55 | 10 | 277,156 | 1,351 | 11.8 | 1.39 (1.21-1.59) | 0 | 0.79 |  |
| ≥55 | 11 | 253,981 | 3,843 | 12.5 | 1.27 (1.15-1.40) | 0 | 0.84 |  |
| Birth Year | | | | | | | | 0.53 |
| <1925 | 5 | 112,186 | 1,739 | 14.4 | 1.27 (1.13-1.43) | 0 | 0.90 |  |
| 1925-1940 | 9 | 175,989 | 2,463 | 11.4 | 1.41 (1.20-1.65) | 0 | 0.65 |  |
| ≥1940 | 9 | 242,962 | 992 | 11.1 | 1.38 (1.16-1.64) | 4.5 | 0.40 |  |
| Education level^d^ | | | | | | | | 0.85 |
| Low | 7 | 113,592 | 1,452 | 12.1 | 1.30 (1.11-1.53) | 0 | 0.74 |  |
| High | 6 | 209,435 | 1,197 | 10.9 | 1.27 (1.05-1.52) | 0 | 0.62 |  |
| Abbreviation: N, number; HR, hazard ratio; CI, confidence interval | | | | | | | | |
| ^a^ HR refers to a summary estimate of effects based on a random-effects model, adjusted for sex, smoking (ever/never), alcohol consumption (ever/never), and education (low/high) and body mass index (kg/m^2^, continuous) depending on availability in each cohort | | | | | | | | |
| ^b^ P value for heterogeneity within each group | | | | | | | | |
| ^c^ P value for heterogeneity between subgroups | | | | | | | | |
| ^d^ Primary school education or below was defined low education level | | | | | | | | |

Supplementary Table 7. Association Between Family History of Gastric Cancer and Gastric Cancer Mortality by Gastric Cancer Subtypes

| Subgroup | Studies, N | Participants, N | Death of Castric Cancer, N | Family History of Gastric Cancer among Cases, % | HR (95%CI)^a^ | Heterogeneity within Subgroups^b^ | | Heterogeneity between Subgroups^c^ |
| --- | --- | --- | --- | --- | --- | --- | --- | --- |
|  |  |  |  |  |  | I^2^ value (%) | *p*-value |  |
| **Total** |  |  |  |  |  |  |  |  |
| Anatomic subsite^d^ | |  |  |  |  |  |  | 0.53 |
| Cardia | 7 | 526,893 | 950 | 8.8 | 1.31 (1.19-1.44) | 0 | 0.99 |  |
| Non-cardia | 7 | 529,798 | 3,855 | 13.7 | 1.39 (1.19-1.62) | 0 | 0.71 |  |
| Histological subtype^e^ | |  |  |  |  |  |  | 0.52 |
| Intestinal | 3 | 526,180 | 177 | 14.1 | 1.55 (1.02-2.36) | 0 | 0.83 |  |
| Diffuse | 3 | 526,042 | 99 | 8.1 | 1.17 (0.56-2.42) | 0 | 0.86 |  |
| **Male** |  |  |  |  |  |  |  |  |
| Anatomical subsite^d^ | |  |  |  |  |  |  | 0.386 |
| Cardia | 7 | 240,796 | 514 | 9.5 | 1.44 (1.07-1.94) | 0 | 0.71 |  |
| Non-cardia | 7 | 242,941 | 2,669 | 12.7 | 1.25 (1.11-1.40) | 0 | 0.60 |  |
| Histological subtype^e^ | |  |  |  |  |  |  | 0.923 |
| Intestinal | 3 | 240,482 | 200 | 10.5 | 1.59 (1.00-2.51) | 0 | 0.63 |  |
| Diffuse | 3 | 240,332 | 50 | 10.0 | 1.51 (0.59-3.83) | 0 | 0.84 |  |
| **Female** |  |  |  |  |  |  |  |  |
| Anatomical subsite^d^ | |  |  |  |  |  |  | 0.536 |
| Cardia | 7 | 286,097 | 436 | 8.0 | 1.34 (0.95-1.89) | 0 | 0.96 |  |
| Non-cardia | 7 | 286,857 | 1,196 | 15.6 | 1.51 (1.29-1.77) | 0 | 0.58 |  |
| Histological subtype^e^ | |  |  |  |  |  |  | 0.396 |
| Intestinal | 3 | 285,698 | 37 | 10.8 | 1.74 (0.61-4.96) | 0 | 0.78 |  |
| Diffuse | 3 | 285,710 | 49 | 6.1 | 1.07 (0.26-2.84) | 0 | 0.89 |  |
| Abbreviation: N, number; HR, hazard ratio; CI, confidence interval | | | | | | | | |
| ^a^ HR refers to a summary estimate of effects based on a random-effects model, adjusted for sex, smoking (ever/never), alcohol consumption (ever/never), and education (low/high) and body mass index (kg/m^2^, continuous) depending on availability in each cohort | | | | | | | | |
| ^b^ P value for heterogeneity within each group | | | | | | | | |
| ^c^ P value for heterogeneity between subgroups | | | | | | | | |
| ^d^ Anatomical subsites by International Classification of Diseases (ICD); cardia (ICD-9: 151.0, ICD-10: C16.0), non-cardia (ICD-9: 151.1-151.6, 151.8-151.9, ICD-10: C16.1-16.6, 16.8-16.9) | | | | | | | | |
| ^e^ Lauren classification by ICD for Oncology 3: intestinal type (8012, 8021, 8022, 8031, 8032, 8046 ,8050, 8082, 8143, 8144, 8201, 8210, 8211, 8220, 8221, 8255, 8260, 8261, 8262, 8263, 8310, 8323, 8480, 8481, 8510, 8512, 8570, and 8576), diffuse type (8020, 8041, 8044, 8141, 8142, 8145, 8490, and 8806) | | | | | | | | |

Supplementary Table 8. Association Between Family History of Gastric Cancer and Gastric Cancer Mortality Grouping According to the Relation of Family History

| Subgroup | Studies, N | Participants,  N | Death of Gastric Cancer, N | HR (95%CI)^a^ | Heterogeneity  within Subgroups^b^ | | Heterogeneity between Subgroups^c^ |
| --- | --- | --- | --- | --- | --- | --- | --- |
|  |  |  |  |  | I^2^ value (%) | *p*-value |  |
| **Family relation** | |  |  |  |  |  | 0.63 |
| Parents | 3 | 137,835 | 48 | 1.30 (1.00-1.70) | 0 | 0.97 | 0.25 |
| Father | 3 | 134,644 | 32 | 1.44 (1.05-1.99) | 0 | 0.90 |  |
| Mother | 2 | 131,622 | 16 | 1.12 (0.71-1.77) | 0 | 0.74 |  |
| Sibling | 2 | 130,700 | 17 | 1.63 (1.05-2.55) | 0 | 0.92 |  |
| Brother | 2 | 129,835 | 12 | 1.93 (1.16-3.22) | 0 | 0.91 |  |
| **Family relations by participant’s sex** | | | |  |  |  |  |
| Male participant |  |  |  |  |  |  | 0.59 |
| Father | 3 | 96,500 | 25 | 1.38 (0.96-1.96) | 0 | 0.68 |  |
| Mother | 2 | 94,554 | 14 | 1.02 (0.61-1.70) | 0 | 0.93 |  |
| Brother | 2 | 93,195 | 10 | 1.19 (1.14-3.43) | 0 | 0.81 |  |
| Abbreviation: N, number; HR, hazard ratio; CI, confidence interval | | | | | | | |
| ^a^ HR refers to a summary estimate of effects based on a random-effects model, adjusted for sex, smoking (ever/never), alcohol consumption (ever/never), and education (low/high) and body mass index (kg/m^2^, continuous) depending on availability in each cohort. | | | | | | | |
| ^b^ P value for heterogeneity within each group | | | | | | | |
| ^c^ P value for heterogeneity between subgroups | | | | | | | |
| Results of gastric cancer deaths of less than 10 cases are not shown in the table. | | | | | | | |

Supplementary Reference

1. Sado J, Kitamura T, Kitamura Y, Zha L, et al.Three-Prefecture Cohort Study Group. Rationale, design, and profile of the Three-Prefecture Cohort in Japan: A 15-year follow-up. J Epidemiol. 2017 Apr;27(4):193-199.
2. Tamakoshi A, Ozasa K, Fujino Y, et al. Cohort profile of the Japan Collaborative Cohort Study at final follow-up. J Epidemiol. 2013;23(3):227-32
3. Akira F, Yoshitaka T, Shoko K, et al. A cohort Study on the Relation of Lifestyle, Personality and Biologic Markers to Cancer in Miyagi, Japan : Study Design, Response Rate and Profiles of the Cohort Subjects. J Epidemiol 5 153-157, 1995
4. Watanabe S, Tsugane S, Sobue T, et al. Study Design and Organization of the JPHC Study. Journal of Epidemiology 2001;11(6sup):3-7.
5. Qiao YL, Dawsey SM, Kamangar F, et al. Total and cancer mortality after supplementation with vitamins and minerals: follow-up of the Linxian General Population Nutrition Intervention Trial. J Natl Cancer Inst. 2009 Apr 1;101(7):507-18.
6. Shu X-O, Li H, Yang G, et al. Cohort Profile: The Shanghai Men’s Health Study. International Journal of Epidemiology 2015;44(3):810-818.
7. Zheng W, Chow W-H, Yang G, et al. The Shanghai Women's Health Study: Rationale, Study Design, and Baseline Characteristics. American Journal of Epidemiology 2005;162(11):1123-1131.
8. Yoo KY, Shin HR, Chang SH, et al. Korean Multi-center Cancer Cohort Study including a Biological Materials Bank (KMCC-I). Asian Pac J Cancer Prev 2002;3(1):85-92.
9. Oh J-K, Lim MK, Yun EH, et al. Cohort Profile: Community-based prospective cohort from the National Cancer Center, Korea· International Journal of Epidemiology 2015:dyv302.
